# Effect of Growth Factor-Loaded Acellular Dermal Matrix/MSCs on Regeneration of Chronic Tympanic Membrane Perforations in Rats

**DOI:** 10.3390/jcm10071541

**Published:** 2021-04-06

**Authors:** Gwang-Won Cho, Changjong Moon, Anji Song, Karthikeyan A. Vijayakumar, Mary Jasmin Ang, Chul Ho Jang

**Affiliations:** 1 Department of Biology, College of Natural Science, Chosun University, Gwangju 61452, Korea; gwcho@chosun.ac.kr; 2 BK21 FOUR Education Research Group for Age-Associated Disorder Control Technology, Department of Integrative Biological Science, Chosun University, Gwangju 61452, Korea; dkswl511@naver.com (A.S.); cartkn1991@gmail.com (K.A.V.); 3 Department of Veterinary Anatomy, College of Veterinary Medicine and BK21 FOUR Program, Chonnam National University, Gwangju 61186, Korea; moonc@chonnam.ac.kr (C.M.); ang.maryjasmin@gmail.com (M.J.A.); 4 Department of Otolaryngology, Medical School, Chonnam National University, Hakdong 8, Dongku, Gwangju 61452, Korea

**Keywords:** tympanic membrane, chronic perforation, acellular dermal matrix, epidermal growth factor, basic fibroblast growth factor, regeneration

## Abstract

The success rate of grafting using acellular dermal matrix (ADM) for chronic tympanic membrane was reported in previous studies to be lower than fascia or perichondrium. Combining mesenchymal stem cells (MSCs) and growth factor-loaded ADM for the regeneration of chronic TMP has not been reported so far. In this study, we hypothesized that combining growth factor-loaded ADM/MSCs could promote the recruitment of MSCs and assist in TMP regeneration. We evaluated the regeneration and compared the performance of four scaffolds in both in vitro and in vivo studies. MTT, qPCR, and immunoblotting were performed with MSCs. In vivo study was conducted in 4 groups (control; ADM only, ADM/MSC, ADM/MSC/bFGF, ADM/MSC/EGF) of rats and inferences were made by otoendoscopy and histological changes. Attachment of MSCs on ADM was observed by confocal microscopy. Proliferation rate increased with time in all treated cells. Regeneration-related gene expression in the treated groups was higher. Also, graft success rate was significantly higher in ADM/MSC/EGF group than other groups. Significant relationships were disclosed in neodrum thickness between each group. The results suggest, in future, combining EGF with ADM/MSCs could possibly be used as an outpatient treatment, without the need for surgery for eardrum regeneration.

## 1. Introduction

Chronic tympanic membrane perforation (TMP) accompanied by chronic otitis media, is a common disease in the field of otology. Common causes are trauma, otitis media, or persistent perforation after the insertion of ventilation tube. Unlike the case of acute TMP, natural closure is quite rare in chronic TMP. Repeated inflammation in patients with TMP may lead to complications such as oomostoiditis and labyrinthine fistula. Therefore, tympanoplasty should be performed as early as possible, while the mastoid cavity is still intact.

To perform tympanoplasty for the repair of TMP, various autologous materials such as temporalis fascia, perichondrium, or cartilage graft have been used depending on the surgeon’s choice [1,2]. Although there are several different graft materials available, each has its own limitations. There is a possible association between donor-site morbidity of autologous graft, privation of graft material during case revision, longer operative times and craniofacial pain secondary to harvesting temporalis fascia, especially during mastication [3]. Advances in material engineering and tissue engineering have led to scaffolds replacing autologous tissue in eardrum regeneration research, and are expected to become a part of outpatient treatment, potentially replacing tympanoplasty. This approach should be implemented in the operating room in the future [4,5,6,7,8,9,10,11].

Among alternative autologous materials for TMP, acellular dermal matrix (ADM) has been used for tympanoplasty [12]. ADM is a dermal matrix without cells, produced by a decellulization procedure, and obtained from a human cadaver or tissue of porcine or bovine origin. ADM is commonly used for wound healing because of its 3D porous structure with sufficient elasticity and adhesion. Moreover, ADM is immunologically stable due to nonimmunogenicity bestowed by the removal of epidermal and dermal cells, which can cause rejection [13].

A limitation of the application of ADM is that it does not contain cells and cannot ensure the accurate positioning and distribution of cells from the surrounding tissue. Therefore, further research is needed on clinical tissue increment of ADM. Previous reports showed that the graft success rate using ADM for chronic TMP in chinchilla was lower than fascia or perichondrium, 78% by DW Laidlaw et al. [14] and 80% (8/10) by McFeely et al. [15].

For enhancing the healing of chronic TMP, use of biomolecules such as epidermal growth factor (EGF) or basic fibroblast growth factor (bFGF) has already been studied [16,17,18,19,20,21,22,23]. Although use of stem cells for this purpose has been infrequently reported compared to biomolecules, stem cells have also shown great promise for enhancement of chronic TMP regeneration [24,25,26,27]. It is not easy to engraft the MSCs alone to the injury site [28]. Several bioscaffolds have therefore been used, alone or in combination with cells, to enhance the regeneration of chronic TMP. ADM is more stable compared to gel foam when it is combined with growth factors or MSCs.

By Hakuba et al.’s clinical study using bFGF with collagen scaffold for chronic TMP regeneration, initial graft success rate was fair, but the recurrence rate of TMP was 18% per year [29,30]. In 15% of neodrum revealed, progressive atrophic TM has been reported [29]. Although no exact mechanisms have been identified, it has been explained that it is due to collagen accumulation inhibitory effects [29]. The possible mechanism of atrophic TM is inhibition of collagen deposition, which affords a minimal benefit and might even be detrimental, causing atrophy of the eardrum and reperforation. 

To overcome this limitation of single use of ADM or bFGF with collagen scaffold, combining MSCs with ADM appears to be a promising method. To date, use of MSCs and growth factors loaded with ADM for regeneration of chronic TMP has not been reported. In this study, we hypothesized that combined use of EGF or bFGF loaded with ADM/MSCscan promote MSC recruitment, and regenerate TMP better than ADM only. The amount of bFGF or EGF can be reduced when scaffold is combined with MSCs, which has the potential for wound healing. The incidence of atrophic change of neodrum may be reduced. The purpose of this study is to evaluate the enhancement of regeneration of chronic TMP, using combined use of ADM/MSC/EGF or bFGF in in vitro and in vivo studies in a rat chronic TMP model. 

## 2. Materials and Methods

### 2.1. In Vitro Study 

#### 2.1.1. Cell Culture and Treatment

Human bone marrow mesenchymal stem cells (MSCs; Cefobio, Korea) were cultured in DMEM growth medium containing low glucose (Gibco; USA), supplemented with 10% fetal bovine serum (FBS; Gibco), 1% penicillin and streptomycin (Lonza), and incubated at 37 °C in humidified atmosphere containing 5% CO_2_. Cells were harvested using 0.25% trypsin-EDTA (Gibco). Harvested cells were then seeded on a 100 mm cell culture dish with flash conditioned media. Cells were counted and 3 × 10^4^ cells were seeded on each 100 mm dish. Seventh passage MSCs (CEFO, South Korea) were used in this study.

1 × 10^4^ MSCs were seeded over acellular dermal matrix (ADM; L&C Seongnam-si, Korea) (1 cm × 1 cm) in a 24-well plate, and further cultured for 24 h in the above-mentioned optimal conditions. MSCs were then treated with 100 ng/mL recombinant human EGF (Gibco) or 100 ng/mL recombinant human basic FGF (Gibco) for 24 h, washed twice with PBS, and used for further experiments.

#### 2.1.2. Live and Dead Cell Staining

Each group of cells was stained for 20 min with Hoechst33342 (dilution 1:1000; Invitrogen), which stains the intact nucleus of live cells, and Propidium iodine (dilution, 1:1000; Invitrogen), which stains the dead cells. A confocal microscope (Leica TCS SP5 AOBS/Tandem, Korea Basic Science Institure, Daejeon, South Korea) was used to capture the images.

#### 2.1.3. Cell Viability Assay 

Treated MSCs were cultured for 1, 3 and 5 days (the time including 24 h treatment of EGF or bFGF). The cells were detached from the ADM using trypsin and the cell viability was assessed by MTT analysis in a 96-well plate. A total of 0.5 mg/mL MTT (3-(4,5-dimethylthiazol-2-yl)-2,5-diphenyltetrazolium bromide; Sigma) was added to the cells, and incubated for 2 h at 37 °C. Formazan was dissolved in DMSO (Sigma). The absorbance value was measured in an ELIZA plate reader (Thermo Scientific, Vantaa, Finland) at 560 nm.

#### 2.1.4. Real-Time PCR (Quantitative PCR) 

Cultured MSCs were harvested from ADM using 0.25% trypsin-EDTA, and total RNA was extracted using RNAiso reagent (TAKARA, Japan). cDNAs were synthesized using Primescript II 1st strand cDNA synthesis kit (TAKARA, Japan). The reaction mixture consisted of 1.5 μg total RNA, 5 μM Random primers, 1 mM of dNTPs, and reaction buffer. The real-time PCR reaction mixture comprised of Power SYBR Green PCR master mix (Applied Biosystems Inc., Cheshire, UK) and specific primers for each gene, namely human *COL1A1, COL3A1, TGFβ1* and *VEGF* (GenoTech Corp., Daejeon, South Korea; Integrated DNA Technologies Inc., Coralville, IA, USA). The sequences of primers are summarized in Table 1. PCR conditions were as follows: initiation at 95 °C for 10 min, followed by 40 cycles of 15 s at 95 °C, and 1 min in at 60 °C.

#### 2.1.5. Immunoblot Analysis

Total protein from each group of cells was isolated with 20 µL RIPA buffer (Santa Cruz Biotechnology, Santa Cruz, CA, USA) supplemented with inhibitor cocktail (Thermo Fisher Scientific; Waltham, MA, USA), phenylmethyl sulfonyl fluoride (PMSF; Enzo) and sodium orthovanadate (Santa Cruz Biotechnology). The extracted proteins were quantified using BSA assay kit (Thermo Fisher Scientific, IL, USA), and the resulting absorbance value was measured with an ELISA microplate reader (Thermo Fisher Scientific) at 562 nm. An equivalent amount of proteins was loaded in a 12% SDS-polyacrylamide gel, and was separated by electrophoresis. The proteins were then electrically transferred onto a PVDF transfer membrane (GE Healthcare, Buckinghamshire, UK). The membrane was blocked using 5% normal horse serum in TBS buffer containing 0.1% Tween 20 (TBS-T) for 90 min. After blocking, the membrane was washed, and pan-cytokeratin primary antibody (dilution 1:200; Santa Cruz Biotechnology) was added and incubated in 4 °C overnight. The membrane was washed again with TBS-T buffer, and horseradish peroxidase conjugated antimouse secondary antibody (dilution 1:2000; Santa Cruz Biotechnology) was added and incubated for 90 min at room temperature. The protein expression was detected using enhanced chemiluminescence (ECL) detection system (Amersham) and exposed to X-ray film (Agfa).

### 2.2. In Vivo Study

#### 2.2.1. Creation of Chronic TMP 

Experiments were performed in 27 male Sprague–Dawley rats (weighing 250 g each, Samtaco Bio Korea, Osan, Korea) with normal tympanic membranes (TM) and Preyer’s reflexes. This study was approved by the animal ethics committee (CIACUC2020-A0018). The rats were brought up in rooms having a constant temperature of 22 °C, humidity of 50%, and an ambient noise level < 40 decibels (dB).

In vivo study was conducted in 4 groups (Control; ADM only, ADM/MSC, ADM/MSC/bFGF, ADM/MSC/EGF) of rats with different scaffolds

**Figure 1:**
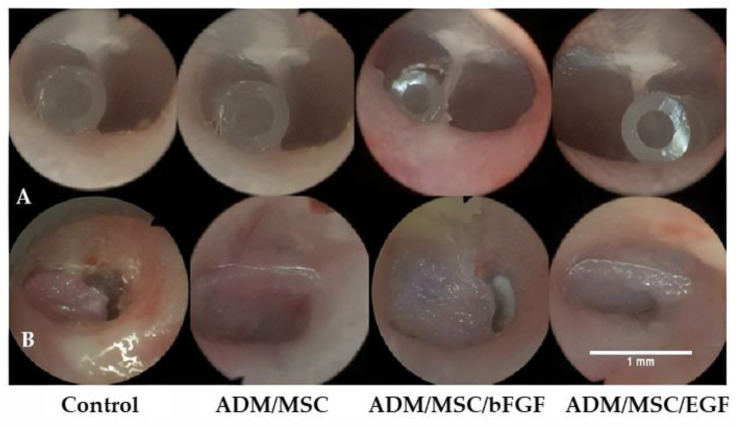
Otoendoscopy shows well patent ventilation tube (angiocatheter). Three groups: left anterior area, acellular dermal matrix/mesenchymal stem cell/epidermal growth factor (ADM/MSC/EGF) group: right anterior (**A**). Otoendoscopy shows the placement of each scaffold (3 mm diameter). They are placed fine in an overlay fashion (**B**).

Only the left TM of control group and ADM/MSC group underwent myringotomy, while ADM/MSC/bFGF group experiment was conducted on both eardrums, because the distribution of eardrum EGF or bFGF receptors differed in populations. Under inhalation anesthesia using isoflurane, the TMs were examined under an operating microscope using black plastic speculum. If otitis media was found, they were excluded. For creating chronic perforation, we modified the method used by Wang et al. [31,32]. A mini angiocatheter (Medicut, 24G, KOREAVACCINE co, Ansan, Korea) made by cutting a long catheter into 1 cm pieces was inserted into the anterior area of the TM (Figure 1A). Following this, a mixed solution containing mitomycin C (0.5 mg/mL, Sigma-Aldrich, Seoul, Korea) and dexamethasone was topically applied for 3 weeks. The minicatheter was removed at 3 weeks postinsertion. At the anterior area of the pars tensa, TMPs sizing to 70% were created by visualization under a surgical microscope. The perforation margin was covered by epithelial layer. Topical application of ciprofloxacin-dexamethasone was extended for 2 weeks.

#### 2.2.2. Scaffold Preparation

ADM was punched out using a 3 mm biopsy punch (Kai Medical Co., Tokyo, Japan). 1 × 10^4^ MSCs were seeded over the round ADM on a 24-well plate and further cultured for 24 h in optimal conditions. MSCs were then treated with 100 ng/mL recombinant human EGF (Gibco), or 100 ng/mL recombinant human basic FGF (Gibco) for 24 h, washed twice with PBS, and used for further experiments.

#### 2.2.3. Application of Scaffold

The rats were subdivided into 4 groups: one control group (ADM only, *n* = 9, left side) and three experimental groups, namely ADM/MSC, *n* = 9, left side, ADM/MSC/bFGF, *n* = 9, right side, and ADM/MSC/EGF, *n* = 9, left side. In this study, ADM/MSC/bFGF and ADM/MSC/EGF were implemented on the same set of rats, as each rat may have a different growth factor receptor distribution [33]. After trimming of the perforation edges by sharp pick and alligator cup forceps, each scaffold was applied in an overlay fashion (Figure 1B). After application of each scaffold, the external auditory canal was packed using fibrin glue.

#### 2.2.4. Otoendoscopic Examination

Every week, each TM was observed through otoendoscopy under inhalation anesthesia, to inspect the healing process. Transcanal digital images were recorded using iPhone attached endoscope (ID 2.7 mm).

#### 2.2.5. Histopathological Examination

Animals were euthanized and decapitated at 4 weeks after scaffold application, and bulla was extracted immediately by dissection. The bony wall of the bulla was perforated by dental root elevator, and bulla was fixed with 10% neutral buffered formalin for 24 h. After decalcification using ethylenediaminetetraacetic acid (EDTA) solution for 7 days, the surrounding bone over the tympanic membrane was trimmed under a surgical microscope. Serial dehydration of the decalcified TM, including the bony annulus, was performed using alcohol, followed by paraffin embedding. The sections were stained using hematoxylin-eosin (HE).

#### 2.2.6. Immunohistochemistry of Ki-67 in the Rat Tympanic Membrane

Rat tympanic membrane samples were processed and sectioned into paraffin embedded tissue sections of 5 µM thickness. Tissue sections were then deparaffinized using routine protocols. Using 0.3% (*v*/*v*) hydrogen peroxide for 20 min, the endogenous peroxidase activity of the tissue was blocked. This was followed by blocking of nonspecific reactions for 1 h using 5% (*v*/*v*) NGS (Vector ABC Elite Kit; Vector Laboratories, Burlingame, CA, USA) in 0.3% (*v*/*v*) Triton X-100. The sections were then incubated with rabbit anti-Ki67 (1:500; Acris, Hiddenhausen, Germany), overnight at 4 °C in an antibody dilution buffer (Invitrogen). Biotinylated goat antirabbit immunoglobulin G (IgG) (Vector ABC Elite Kit; Vector Laboratories) was incubated at RT for 1 h to detect primary antibody binding. Immunoreactivity was measured using an avidin–biotin peroxidase complex (Vector ABC Elite Kit; Vector Laboratories), which was incubated for 1 h at RT according to the manufacturer’s instructions. The peroxidase reaction was carried out with diaminobenzidine substrate (contained in the DAB kit; Vector Laboratories) assay based on the manufacturer’s instructions. In a few test sections in each experiment, primary antibodies were omitted. The slides were counterstained with Harris’ hematoxylin, before being mounted with Canada balsam (Sigma-Aldrich).

### 2.3. Statistical Analysis

Statistical analysis was performed using Prism software (v 8.8; GraphPad). Data of the TMP regeneration rates by otoendoscpoy were compared with Kruskal–Wallis test. One-way ANOVA followed by Tukey post hoc test was used to analyze the in vitro test data. The data were considered statistically significant when the *p* value was less than 0.05.

## 3. Results

### 3.1. In Vitro Study 

**Figure 2:**
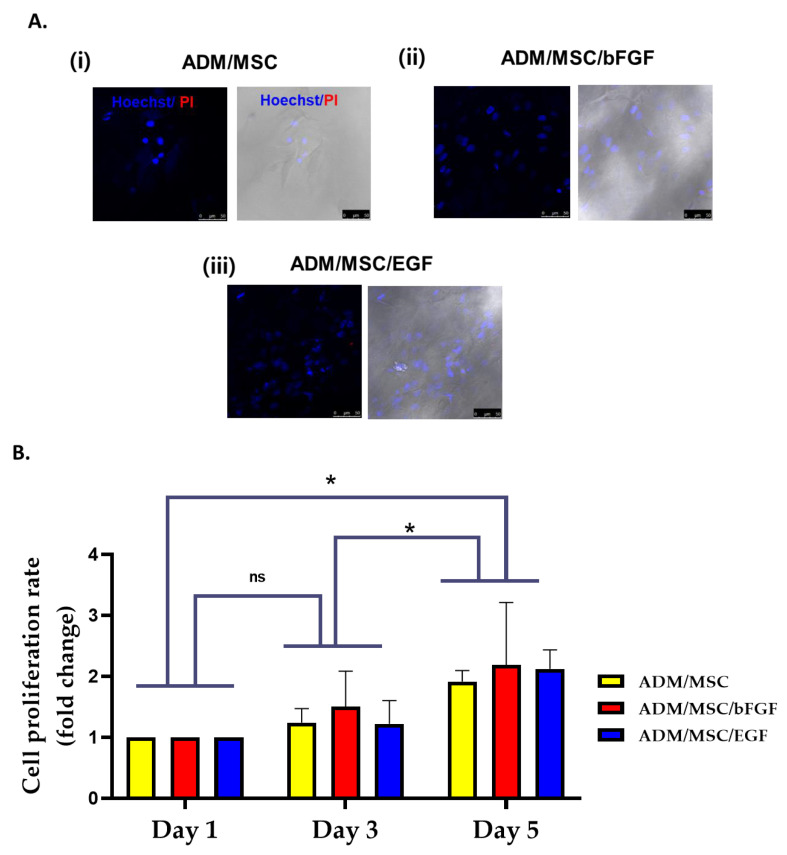
(**A**) Live and dead cell staining of hBM-MSCs on acellular dermis observed under confocal microscope. Increased number of cells was observed in each group treated with (ii) basic fibroblast growth factor (bFGF) and (iii) epidermal growth factor (EGF), than in the (i) control group. (**B**) The proliferation rate of hBM-MSCs through MTT assay was confirmed after 1st, 3rd and 5th days culture. Number of cells were increased the most in AC/MSCs/EGF group. Cell proliferation was increased in a time-dependent manner in all the groups. (*n* = 3). Significant difference in the proliferation rate was not observed between Day1 and Day3, but there was a significant change in the fold change between Day1/Day5 and Day3/Day5 (* *p* < 0.05); ns = non significant.

MSCs were seeded on acellular dermal matrix (ADM + MSCs) containing growth media, and treated with EGF (ADM/MSCs/EGF) or bFGF (ADM/MSCs/bFGF) for 24 h. The cells were well attached and no dead cells were observed in any of the designed groups (Figure 2A(i)–(iii)). Number of cells was seen increased in the growth factors treated groups (Figure 2A(ii)–(iii)). Cell viability was measured in additional cultures for 1, 3 and 5 days after the treatment of EGF or bFGF. Cell viability increased in a time-dependent manner in all treated cells (Figure 2B.).

**Figure 3:**
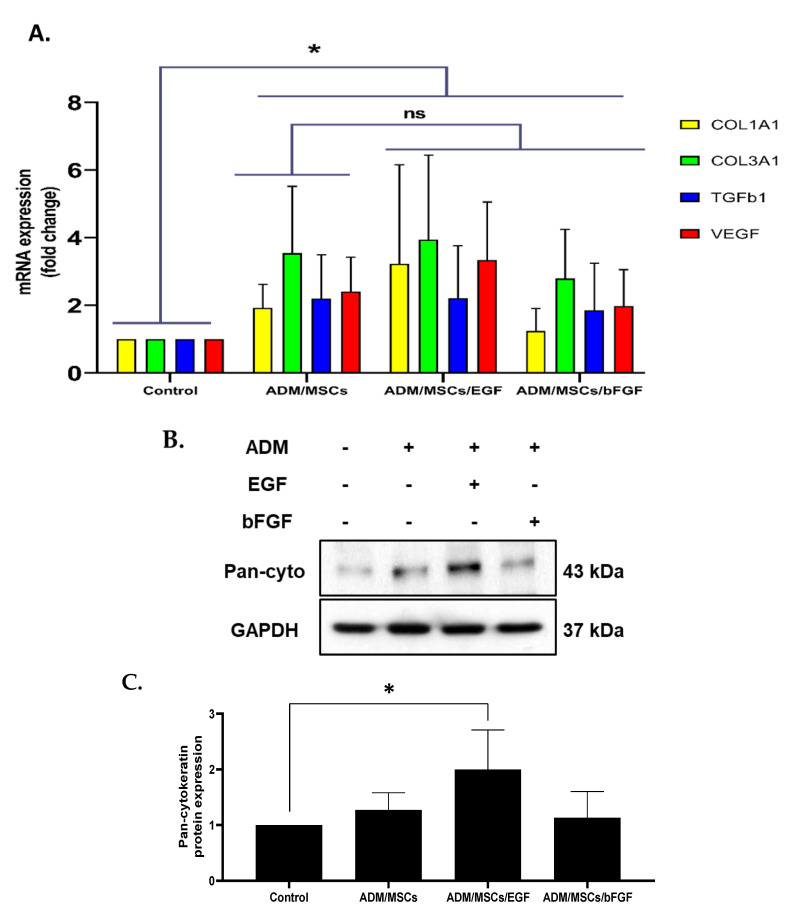
(**A**) Expression levels of mRNA markers for tympanic membrane regeneration. The gene expression of Col1A1, Col3A1, TGFb1 and VEGF in the experimental group was significantly higher than that of the control group (ADM). Although, there was no statistical significance, EGF treated group (ADM/MSC/EGF) showed the highest expression level of collagen and VEGF. COL1A1: Collagen type I alpha 1, COL3A1: Collagen type III alpha 1, TGFb1: Transforming growth factor-beta, VEGF: Vascular endothelial growth factor, (**B**,**C**) Immunoblot showing the expression of pan-cytokeratin. Among three experimental groups, the ADM/MSC/EGF group showed significantly higher expression compared to the control group. Image processing was carried out using Image J. (* *p* < 0.05, *n* = 6), ns = not significant explanation.

To examine the differentiation rate in molecular level, RT-PCR was performed with primers targeting *COL1A1* (collagen, type I, alpha1), *COL3A1* (collagen, type III, alpha1), *TGFβ1* (transforming growth factor beta1) and *VEGF* (vascular endothelial growth factor) genes (Table 1). The expression levels of all of these genes were found increased in three experimental groups when compared with the control group (Figure 3A). Expression of pan cytokeratin was checked with immunoblotting using Pan-cytokeratin specific antibody. Among three experimental groups, the ADM/MSC/EGF group showed significantly higher expression compared to the control group (Figure 3B,C).

### 3.2. In Vivo Study

#### 3.2.1. Otoendoscopic Examination

**Figure 4:**
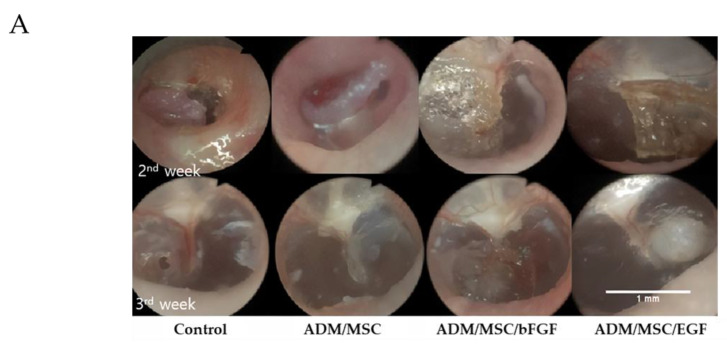

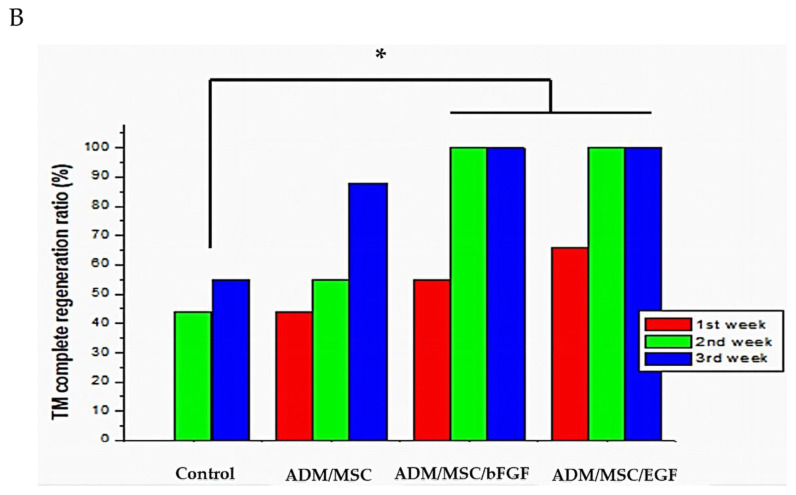
(**A**) Regeneration of Tympanic membrane. Otoendoscopy (2nd week) showed that control or ADM/MSC group causes no interval change, but overlayed scaffold in the ADM/MSC/bFGF group or ADM/MSC/EGF group findings show progression to more advanced state. Otoendoscopy (3rd week) revealed a persistent, small perforation in the control group, but intact neodrum was achieved in other groups. Among three groups, ADM/MSC/EGF group showed prominent epithelization of neodrum. (**B**) The graphic photo showed the tympanic membrane regeneration ratio of different groups. Both ADM/MSC/bFGF group and ADM/MSC/EGF group showed significantly increased regeneration rate when compared with control group. (* *p* < 0.05).

All rats survived well without any infection or complication after scaffold application. Complete TM regeneration rate was achieved in both the groups; 88.8% in ADM/MSC/bFGF group, and 100% in ADM/MSC/EGF group within the 2nd week. But only 6 out of 9 (66.6%) rats in ADM/MSC group, and 4 out of 9 (44.4%) rats in the control group, showed TM regeneration. Successful chronic TMP regeneration was achieved the most in ADM/MSC/EGF group, followed by ADM/MSC/bFGF group, ADM/MSC group, and the least in the control group. Lowest success rate of TMP regeneration was observed in the control group, that is, 5 out of 9 rats (55.5%). However, the experimental groups showed complete TM regeneration within 3rd week. (Figure 4A,B). Healing time of ADM/MSC/bFGF group and ADM/MSC/EGF group was faster when compared to that of control group and ADM/MSC group. This difference in TMP regeneration was statistically significant in ADM/MSC/bFGF group and ADM/MSC/EGF group, compared to ADM/MSC group and control group. In otoendoscopy, neodrum was found to be thicker in ADM/MSC/bFGF group and ADM/MSC/EGF group than in control group and ADM/MSC group. Grossly, more thickened neodrum was found in ADM/MSC/bFGF group and ADM/MSC/EGF group, when compared to control group and ADM/MSC group. Especially, prominent regeneration of the neodrum was noted in ADM/MSC/EGF group (Figure 4A,B). 

#### 3.2.2. Histological Results

**Figure 5:**
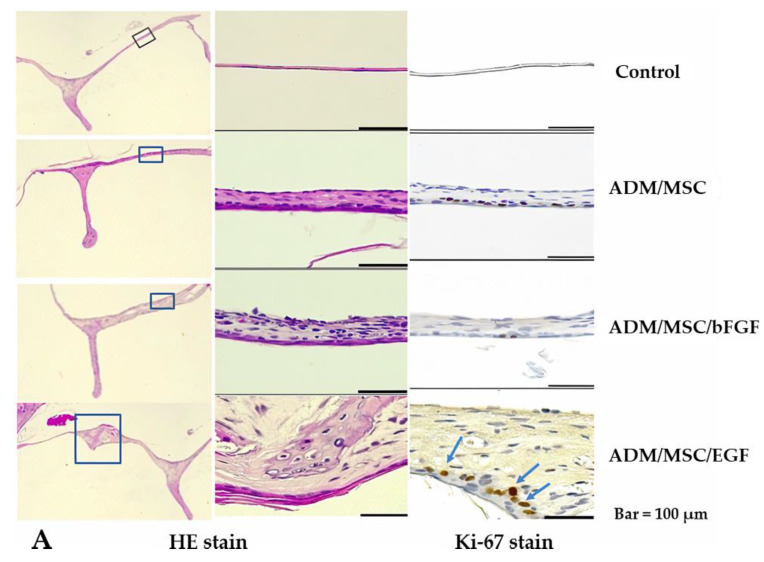

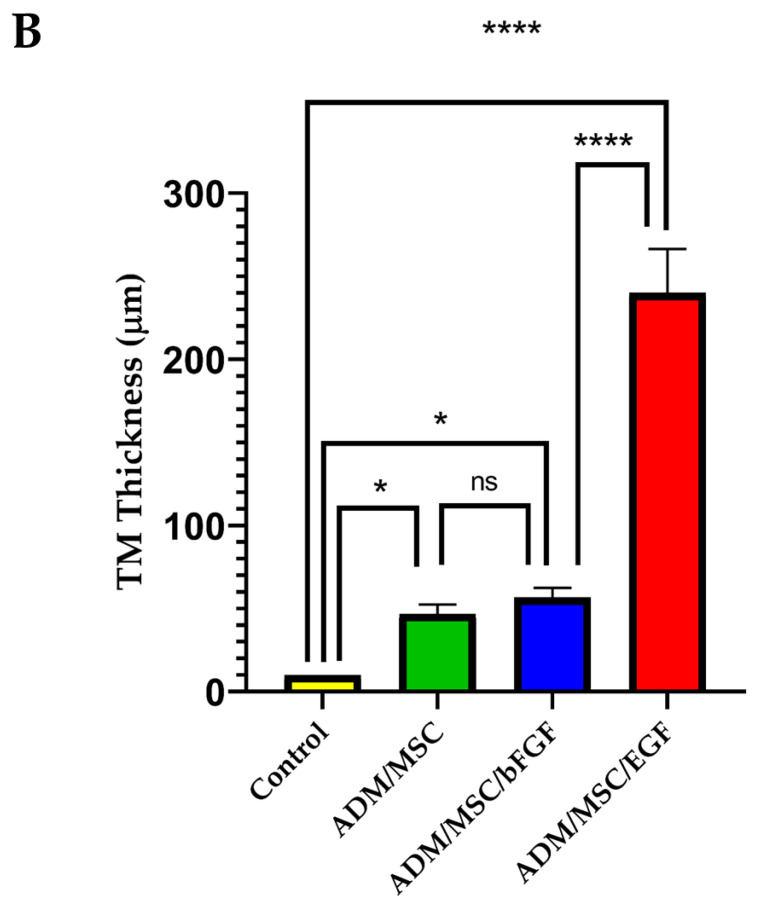
(**A**) HE stain showed the neodrum in each group. Compared to the control group, other groups showed greater thickening of neodrum in TM regeneration. Among them, ADM/MSC/EGF group revealed the most thickened neodrum. Staining for Ki-67, a proliferation marker, shows keratinocyte formation in the epithelial layer. The ADM/MSC/EGF group showed keratinocyte formation in the epithelial layer (arrows). (**B**) The mean thickness of the neodrum in each group was analyzed by ANOVA test with Tukey post hoc multicomparison test. * *p* < 0.05, **** *p* < 0.0001, ns = not significant explanation.

Histological examinations showed that regenerated neodrum healed well. The neodrum was focally thicker in ADM/MSC/bFGF group and ADM/MSC/EGF group, compared to control group and ADM/MSC group. The fibrous layer was prominently regenerated (Figure 5A,B). The expression of ki-67, a proliferation marker, showed the same results as HE staining, whose expression was prominent in ADM/MSC/EGF group, compared to control group or ADM/MSC group. A large number of Ki-67-positive cells was observed at the base (arrows) of the epidermal layer. 

## 4. Conclusions 

In this study, we investigated the enhancing effect of MSC-loaded ADM with EGF or bFGF on regeneration of perforated tympanic membrane. Our results from both in vitro and in vivo studies showed that the ADM/MSC/EGF group had a more enhancing effect compared to ADM/MSC/bFGF group.

ADM is an allograft dermal matrix that is chemically processed into a decellularized bioactive matrix by the removal of all epidermal and dermal cells. ADM contains extracellular matrix such as collagen and elastin, on which the surrounding cells adhere. They also act as a stable scaffold for cell regeneration. In this study, confocal fluorescence microscopy revealed that MSCs were well attached to, and stayed in the ADM scaffold. The disadvantages of ADM are inadequate revascularization and high cost [34].

Generally, in the process of wound healing after myringotomy, the epithelial layer is the first to close the TM. For this wound healing process, cell proliferation, migration and higher metabolic activity are required. Though the epithelial layer is the first to close the TM, the events in the other layers are equally important. During cell migration and proliferation, the epithelial layer shows increased metabolic activity, requiring a massive supply of nutrients and oxygen [35]. Secondary processes include increase in vascularization and angiogenesis in the lamina propria [36]. In this study, in vitro real time PCR results showed increased expression of mRNAs of VEGF in MSC or EGF, bFGF loaded scaffold in ADM/MSC group, ADM/MSC/bFGF group and ADM/MSC/EGF group, respectively. Therefore, we conclude that MSC or loaded EGF or bFGF enhanced vascularization and angiogenesis in the lamina propria, through the production of VEGF mRNA. To the extent that it cannot be overemphasized, the role of VEGF is important in tissue regeneration.

In this study, we used the bone marrow derived MSC, which is known to secrete VEGF by paracrine action and promote wound healing by recruiting macrophages and vascular endothelial cells at the wounded region [37]. Goncalves et al. [38] and Duscher et al. [39] suggested multiple mechanism of MSCs in wound healing of TMP, including direct differentiation of the progenitor cells in the perforation margin, modulation of inflammation, secretion of trophic factors and other paracrine effectors such as vascular endothelial growth factor, matrix metalloproteinase-9, EGF or keratinocyte growth factor and insulin-like growth factor. In this study, we could not observe the inflammatory findings by otoendoscopic findins or histological findings.

Our real-time PCR results showed that expression of the COL1A1, COL3A1, TGFβ1 and VEGF genes increased in all treated groups. In the in vitro study, MTT analysis showed that cell viability was the highest in ADM/MSC/EGF, and the expression of COL1A1, COL3A1, and VEGF genes was also the highest in ADM/MSC/EGF. Immunoblotting of pan-cytokeratin also revealed that the highest levels of the protein were in ADM/MSC/EGF, compared to other groups. We found that loaded EGF hastened the rate of regeneration of chornic TMP in the in vivo study.

To date, the effect of topical application of EGF on the regeneration of human TMPs has been controversial. Ramsay et al. [40] reported that the use of topical EGF did not affect TMP regeneration, while Lou et al. observed positive effects and reduction of TMP healing time for both acute TMP and subacute TMP [41,42].

Numerous studies have been reported since Hakuba et al. [30]. Kanamaru et al. [43] reported effects on chronic TMP patients using topical bFGF or EGF. To date, ototoxicity has not been reported after topical application of EGF or bFGF for TMP regeneration, in either animals or humans [42,44].

It is equally important to repair the fibrous layer, because most spontaneously regenerated neodrum showed deficient fibrous layer, which appears like a very thin translucent neodrum [45,46].

Collagen is a predominant component of the skin’s connective tissue, and forms the fibrous layer of TM [47]. The fibroelastic structural characteristics of collagen enable it to withstand variations in air pressure, such as barotrauma or tubal dysfunction. This is because of it is weaker than usual structure, caused by the deficient formation of the fibrous layer. In this study, compared to control group, all experimental groups showed increased expression of COL1A1, COL3A1, and TGFβ1 genes in all treated groups. And the in vivo study showed an increase in the thickness of the regenerated epithelial and fibrous layer. Our previous studies showed the recovery of nanovibration of the neodrum using laser Doppler vibrometer [48,49]. Rahman et al. [26] observed a thickened middle fibrous layer in the neodrum caused by abundant fibroblasts and extracellular matrix using electron microscopic examination. The thickened middle fibrous layer in the neodrum is similar to our study. This thickened middle fibrous layer is important in case of Eustachian tube dysfunction.

The combination of ADM with MSC and growth factors can integrate the advantages of each material, and make up for biological defects or limits in mechanical strength. Lack of ADM/EGF or ADM/bFGF group is the only limitation of this study. MSC or growth factor merged with ADM is believed to enhance eardrum regeneration, and can be used as an outpatient treatment without surgery in the future.

## Figures and Tables

**Table 1:** Showing the primer sequences of the genes used for real time PCR.

Gene	Sequence (5′–3′)
*COL1A1*	CTGGAAGAGTGGAGAGTACTGG GGAATCCATCGGTCATGCTCT
*COL3A1*	GAAAGAGGATCTGAGGGCTC CTCCATAATACGGGGCAAAACC
*TGFβ1*	CAAGTGGACATCAACGGGTTC TCCGTGGAGCTGAAGCAATAG
*VEGF*	CTACCTCCACCATGCCAAGTG GCAGTAGCTGCGCTGATAGAC

## Data Availability

The data presented in this study are available on request from the corresponding author.
